# Increasing the Reinforcing Value of Exercise in Overweight Adults

**DOI:** 10.3389/fnbeh.2019.00265

**Published:** 2019-12-03

**Authors:** Kyle D. Flack, Kelsey Ufholz, LuAnn Johnson, James N. Roemmich

**Affiliations:** ^1^Department of Dietetics and Human Nutrition, University of Kentucky, Lexington, KY, United States; ^2^United States Department of Agriculture-Agricultural Research Service (USDA-ARS), Grand Forks Human Nutrition Research Center, Grand Forks, ND, United States

**Keywords:** exercise, motivation, reward, dopamine, incentive sensitization

## Abstract

**Objectives**: This study determined whether a moderate- or high-dose exercise program increases exercise reinforcement. Increasing the relative reinforcing value of exercise (RRV_exercise_; i.e., incentive sensitization of exercise) may increase the usual physical activity (PA) participation. Preference and/or tolerance for the intensity of exercise was also assessed.

**Design**: Sedentary men and women (body mass index, BMI: 25–35 kg/m^2^) were randomized into parallel exercise training groups expending either 300 (*n* = 18) or 600 (*n* = 18) kcal/exercise session, five sessions/week, for 12 weeks.

**Methods**: The RRV_exercise_ was determined by how much work was performed for exercise relative to a sedentary alternative in a progressive ratio schedule task. Preference and tolerance for exercise intensity were determined by questionnaire.

**Results**: RRV_exercise_ increased (*P* < 0.05) in both groups. Exercise reinforcement, defined as the amount of work completed for exercise without taking sedentary activity into account, increased (*P* < 0.01) in the 600 kcal group only. Preference and tolerance for exercise intensity increased (*P* < 0.01) in both groups, which predicted increases in RRV_exercise_.

**Conclusion**: Expending 300 or 600 kcal, 5 days/week increases RRV_exercise_, while 600 kcal, 5 days/week may be needed to increase exercise reinforcement.

## Introduction

Successful adherence to regular exercise remains a challenge for many adults (Tucker et al., 2011; Moore et al., 2012) and there is great interest in the psychological and psychosocial mechanisms influencing exercise participation (Marcus et al., 2000; Marshall and Biddle, 2001; Speck and Harrell, 2003). Cross-sectional work has demonstrated that adults who find aerobic exercise highly reinforcing are more likely to meet physical activity (PA) guidelines for vigorous physical activity (VPA) while those who find resistance-type exercise more reinforcing are more likely to meet recommendations for muscular-strengthening activities and VPA (Flack et al., 2017a). Behavioral Choice Theory provides a framework for understanding the choices people make and how to shift choice from less healthy to healthier alternatives, such as choosing to be physically active rather than sedentary. In the case of PA or exercise, the primary determinants of the choice to exercise over a sedentary behavior include the reinforcing value of exercise relative to other available alternatives (termed relative reinforcing value or RRV) and access to either exercise or alternative sedentary behaviors (Epstein and Roemmich, 2001). If sedentary and physical activities are equally accessible, the difference in reinforcement between sedentary and physical activities determines the choice (Epstein and Roemmich, 2001). The RRV of exercise (RRV_exercise_) is usually low when assessed against competing sedentary activities (RRV_sedendary_; Epstein et al., 1999; Roemmich et al., 2008; Barkley et al., 2009), which tend to be less effortful and may be perceived as more pleasant compared to the physical discomfort of exercise. Understanding the determinants of the RRV_exercise_ and how to increase it may offer insight into shifting choice to increase PA adherence, leading to improved health.

Increasing the reinforcing value of exercise relative to sedentary behavior (RRV_exercise_) should shift choice towards physically active behaviors, increase exercise participation, and result in more Americans meeting PA guidelines. Increasing RRV of a behavior can be accomplished *via* “incentive sensitization,” originally proposed to explain drug addiction (Robinson and Berridge, 1993). According to incentive sensitization theory, the psychological process of incentive salience transforms the perception of stimuli, increasing their salience within the environment to produce a bias of attentional processing towards the stimuli after repeated exposures (Robinson and Berridge, 1993). This produces neuroadaptations that increase the motivating value of the behavior (Epstein and Roemmich, 2001; Robinson and Berridge, 2008). The result is an increased reinforcing value of the stimulus relative to a competing alternative. Incentive sensitization theory has typically been applied to well-known, highly-reinforcing behaviors, including drug abuse, alcoholism, gambling, and eating (Robinson and Berridge, 1993; Epstein et al., 2007; Temple and Epstein, 2012; Robinson et al., 2016). These highly-reinforcing behaviors are products of the central dopamine system, initiating a dopaminergic response to modulate their reinforcing value (Arias-Carrión et al., 2014). Regulation of exercise behaviors by the central dopamine system is not fully elucidated, but evidence from animal models modifying dopamine transporter and receptor expression to influence PA behaviors points to dopamine playing a major role in voluntary PA (Rhodes and Garland, 2003; Bronikowski et al., 2004). Our group has found similar parallels in humans, where single nucleotide polymorphisms (SNPs) involved in central dopamine signaling and implicated in drug abuse reinforcement also influence RRV_exercise_ (Flack et al., 2019). This offers an explanation as to why exercise dependency has been demonstrated in humans (Chan and Grossman, 1988; Chapman and De Castro, 1990; Belke, 1997; Holden, 2001) and that rodents will respond for exercise (Iversen, 1993; Belke, 1997, 2000; Lett et al., 2000), with some arguing that central dopamine is playing a major role in the choice to be physically active (Knab and Lightfoot, 2010). Although exercise and well-established reinforcing behaviors such as drug abuse may not share identical pathways, evidence mentioned above points to both being at least partially controlled by central dopamine signaling. Therefore, lessons learned from drug abuse or literature from other reinforcing behaviors may help inform us of exercise reinforcement and the process of incentive sensitization for exercise. This was the basis of a recent investigation by our group, demonstrating a low-dose exercise intervention to be ineffective at increasing exercise reinforcement and effective at decreasing sedentary behavior reinforcement, which resulted in less sedentary and more light-intensity PA post-intervention (Flack et al., 2019). Furthermore, this study demonstrated that tolerance to exercise discomfort was related to incentive-sensitization for exercise. It is likely that exercise session parameters such as the dose, duration, or intensity of the exercise exposures are important variables in the process of increasing RRV_exercise_. Greater duration of exposure is more effective at increasing drug reinforcement (Wolffgramm and Heyne, 1995; Ahmed and Koob, 1998; Heyne and Wolffgramm, 1998; Deroche-Gamonet et al., 2004; Ferrario et al., 2005). Thus, greater duration and volume of exercise completed during an exercise session may be more effective at increasing RRV_exercise_.

Another factor that may influence exercise behavior and RRV_exercise_ is the preference for and/or tolerance to exercise intensity (Ekkekakis et al., 2008a; Lind et al., 2008; Flack et al., 2017a). Preference for and tolerance to the unpleasant (e.g., muscle pain, breathing hard) aspects of exercise may influence the choice to be physically active and is greater in individuals who meet PA guidelines (Flack et al., 2017a). Individuals with greater tolerance still experience these unpleasant aspects of exercise; however, they are better able to handle them, making it possible for them to find exercise more reinforcing. Preference and tolerance for exercise intensity are associated with the frequency of participation in strenuous exercise and total leisure-time exercise, independent of RRV_exercise_ (Ekkekakis et al., 2008b). Investigations have not yet tested whether the preference or tolerance for exercise intensity can be increased with repeated exposures to exercise or whether increases in preference or tolerance mediate the effects of exercise exposures on the increase in RRV_exercise_. Understanding the factors that influence the incentive sensitization of exercise would yield valuable information that could be used to design exercise programs that improve aerobic fitness while concurrently increasing RRV_exercise_ and long-term adherence to PA recommendations.

Thus, the purpose of the current study was to determine whether engaging in 12-weeks of moderate-dose (five sessions per week at 300 kcal per session, or 1,500 kcal energy expenditure/week) or high-dose (five sessions per week at 600 kcal per session, or 3,000 kcal/week) exercise training produces incentive-sensitization of RRV_exercise_ and whether increases in preference and tolerance for exercise intensity mediate the effects of exercise dosage on increases in RRV_exercise_. It was hypothesized that the intervention group participating in 3,000 kcal of exercise per week would realize greater improvements in RRV_exercise_, which would be mediated by greater increases in preference and tolerance for exercise intensity. The analyses and results presented are secondary outcomes from a study designed to test the compensatory physiological and behavioral responses to increasing exercise energy expenditure.

## Materials and Methods

### Participants

A total of 36 participants (26 females) between the ages of 18 and 49 years volunteered for the study and were randomized into study groups. Of these, 29 participants completed the study (21 females) with six (five females) participants voluntarily withdrawing citing personal reasons. One participant was dismissed for non-compliance with exercise training, defined as either not adhering to the 600 or 300 kcal expenditure prescription ±100 kcal in 90% of their sessions or not completing at least 18 of the prescribed 20 exercise sessions per month (90% completion rate). All participants were inactive (i.e., exercising less than twice per week) with a body mass index (BMI) ranging from 25 to 35 kg/m^2^. Recruitment occurred between April and October of 2016 in the greater Grand Forks, North Dakota metropolitan area. Participants were a sample who responded to recruitment media including printed brochures and flyers and online advertisements placed on the Grand Forks Human Nutrition Research Center website. All participants were non-smokers, not dieting to lose weight, and healthy enough to participate in an exercise program assessed by the Physical Activity Readiness Questionnaire (PAR-Q; Thomas et al., 1992). All participants provided consent and the study was approved by the University of North Dakota Institutional Review Board.

Upon completion of baseline assessments, participants were randomized (1:1 allocation ratio), with allocation concealment, into parallel exercise treatment groups expending 300 or 600 kcal per exercise session, 5 days per week. The random allocation sequence was generated using the Plan procedure in SAS with a block size of four and both treatments randomly occurring twice within each block. The study statistician generated and maintained the allocation sequence and concealed the sequence until the participants were enrolled and interventions were assigned. There was no blinding of assignment to interventions. The trial is registered with ClinicalTrials.gov identifier: NCT02152501.

### Exercise Intervention

Each participant received a set of personalized heart-rate based exercise sessions designed to expend the assigned energy per session (i.e., 300, 600 kcal/session or 1,500, 3,000 kcal/week) based on individual kcal expenditure rates determined from indirect calorimetry during exercise (explained below). Participants were provided two low-intensity steady-state exercise sessions and three interval-based sessions that were of greater intensity each week. To monitor heart rate and record exercise sessions, each participant received a Garmin Vivofit (Kansas City, KS, USA) for the duration of the 12-week intervention. Participants returned to the lab each week to download their workouts and to receive a new set of exercise sessions that only changed in the amount of time spent at different intensities, always resulting in the assigned energy expenditure. After repeating the incremental fitness test at 6 weeks, the average energy expenditure of each heart rate zone was recalculated so new exercise sessions administered thereafter reflected changes in aerobic fitness. To standardize the exercise environment and to prevent access to fitness facilities from presenting a barrier, participants were provided a 12-week pass to a local fitness center upon beginning the exercise intervention. Missed sessions were made-up on subsequent weeks.

### Assessments

#### Relative Reinforcing Value of Exercise and Liking of Exercise

Participants’ reinforcing value of exercise and sedentary behaviors were assessed using their most liked exercise ergometer (elliptical, treadmill, bicycle) or running/walking on an indoor track and their most liked sedentary behavior out of the options presented (watching TV, playing video games, reading magazines, doing crossword puzzles or Sudoku). The RRV_exercise_ was calculated by comparing their reinforcing value of exercise to their reinforcing value of sedentary behaviors (explained below). Participants rated how much they liked each exercise and sedentary activity on a 1–10 scale at baseline and post, with changes in liking calculated by subtracting the baseline score from the post score. The highest liked exercise and sedentary option were used in the reinforcement task. Reinforcing value was determined by measuring the number of responses participants made for exercise or sedentary activities on progressive variable ratio schedules of reinforcement. The task measures how much individuals want to engage in each behavior, a separate construct from liking (Bickel et al., 2000; Epstein et al., 2011; Casperson et al., 2017). The testing environment included two workstations with computers in the same room. One computer was set up for participants to earn points for their highest-liked exercises while the other for their highest-liked sedentary activity. Participants could switch between stations as much as they chose. Participants were instructed on the use of the computer-generated task they engaged in to earn points (equivalent to minutes) toward their most wanted exercise or sedentary activity, or both. The computer task presented a game that mimicked a slot machine; a point was earned each time the shapes matched. After earning five points, a schedule was completed and the participant received 5 min of exercise or 5 min of sedentary activity time depending on what was earned. The game was performed until the participant no longer wished to work for access to either behavior. The schedules of reinforcement were progressive variable ratio (±5%) schedules whereas points were delivered after every four presses initially, but then the schedule of reinforcement doubled [4, 8, 16, 32, (…) 1,024] each time five points were earned (Bickel et al., 2000; Epstein et al., 2011). Participants were awarded the time they earned for each activity after completing the game. The test was conducted in a separate lab within a large fitness center with access to exercise equipment if participants earned exercise time. Sedentary activity time was spent inside the lab, which had televisions and couches. Outcome measures included the breakpoint, or P_max_ (Bickel et al., 2000), which was the last schedule of reinforcement (i.e., 4, 8, 16…) completed for the behavior (exercise or sedentary activity) and RRV_exercise_, which was calculated as [P_max_ exercise/(P_max_ sedentary + P_max_ exercise)] assessed at baseline and post. Outcomes, therefore, include exercise reinforcement (P_max_ exercise), sedentary behavior reinforcement (P_max_ sedentary), and the RRV_exercise_, which takes both of these constructs into account and can be increased by reducing sedentary reinforcement or increasing exercise reinforcement.

#### Preference and Tolerance for Exercise Intensity

Participants’ preference and tolerance for exercise intensity were measured by a questionnaire. Participants completed the validated Preference for and Tolerance of the Intensity of Exercise Questionnaire (PRETIE-Q, Ekkekakis et al., 2005, 2008b) at baseline and post. The PRETIE-Q is an 18-item survey with nine items assessing preference for exercise intensity (i.e., “I would rather work out at low-intensity levels for a long duration than at high-intensity levels for a short duration”) and nine items assessing tolerance for exercise intensity (i.e., “Feeling tired during exercise is my signal to stop or slow down”). These items are summed for each category to produce separate scores for preference for exercise intensity and tolerance for exercise intensity. Summing scores for preference and tolerance results in the “preference and tolerance for exercise intensity” score.

#### Exercise Energy Expenditure

A graded exercise treadmill test was used to determine each participant’s rate of energy expenditure at four different heart-rate zones based on the heart-rate reserve (HRR) method. Resting and exercise heart rate were measured using a Garmin vivofit, which included a chest-strap heart rate monitor similar to a Polar device. Oxygen consumed and expired CO_2_ were analyzed by indirect calorimetry (Oxycon Mobile, CareFusion). Upon completion of a 5-min warm-up walking at 0% grade, 3.0 mph, the treadmill grade increased to 2.5% for 3 min. The treadmill grade was then increased every 3 min to produce an approximate 10 beat per minute increase in heart rate from the previous stage with the speed fixed at 3.0 mph. The test continued until a heart rate of 85% HRR was attained or the participant felt they could no longer continue. Rates of energy expenditure (kcal per minute) at different heart rate zones were calculated from the amount of oxygen consumed and CO_2_ expired using the Weir equation (Weir, 1949) and regressed against heart rate. Energy expenditure was then averaged across each heart rate zone for the determination of energy expenditure per minute per zone for each individual. Heart rate zones (Marcus et al., 2000; Speck and Harrell, 2003; Tucker et al., 2011; Moore et al., 2012) were calculated using HRR as (220-age)-resting HR * zone % + resting HR (Swain et al., 1998). Heart rate zone 1 ranged from 45 to 55%, zone 2 corresponded to 56–65%, zone 3 was 66–75% and zone 4 was 76–85%. Exercise sessions for each participant were prescribed by calculating the amount of time in each zone, or combinations of zones, that would achieve the appropriate energy expenditure (either 300 or 600 kcal). The treadmill test was repeated after 6 weeks to adjust the intensity and duration of the exercise training sessions to account for improvements in aerobic fitness.

#### Anthropometrics, Body Composition

Height was measured in triplicate to the nearest 0.1 cm using a stadiometer (Seca, Chino, CA, USA). Bodyweight was measured using a calibrated digital scale (Fairbanks Scales- Model SCB-R9000-HS; Kansas City, MO, USA) to the nearest 0.1 kg. Measures were completed with participants wearing either provided lab scrubs or light casual clothes (t-shirt, shorts) and not wearing shoes. Body composition was measured using a GE Lunar iDXA machine prior to the exercise test on the same visit. The iDXA technique allows the non-invasive assessment of soft tissue composition by region with a precision of 1–3% (Rothney et al., 2012). A total body scan was conducted with participants lying supine on the table and arms positioned to the side. Most scans were completed using the thick mode suggested by the software, as participants were overweight to obese. All scans were analyzed using GE Lunar enCORE Software (13.60.033). Automatic edge detection was used for scan analyses. The machine was calibrated before each scanning session using the GE Lunar calibration phantom.

### Analytic Plan

Differences in the pre-post changes in Pmax_exercise_, Pmax_sed_, RRV_exercise_, preference, tolerance, and preference + tolerance for exercise intensity were assessed between groups (300 or 600 kcal/session) and if changes were different from zero using analysis of covariance, covarying for baseline values. Sex, age, and percent body fat were considered as additional covariates, but were not significant predictors of any of the outcomes and were not included in the final models. The models were fit with the Glimmix procedure in SAS (SAS Institute Inc., Cary, NC, USA). The Glimmix procedure can be used to fit generalized linear models using a variety of statistical distributions. The beta distribution was used to model RRV, whose values range from 0 to 1, and is therefore not normally distributed. Regression analyses were performed to determine predictors of changes in RRV and Pmax, considering changes in liking (hedonic value) of exercise or changes in preference and tolerance for exercise intensity while covarying for the corresponding baseline value of the predictor and outcome. Although there were no baseline differences between groups for any outcome, covarying for the corresponding baseline value is regarded as the best practice for clinical trials as several other factors can influence results when not covarying for baseline values (Guideline on Adjustment for Baseline Covariates in Clinical Trials, 2015).

## Results

**Figure 1:**
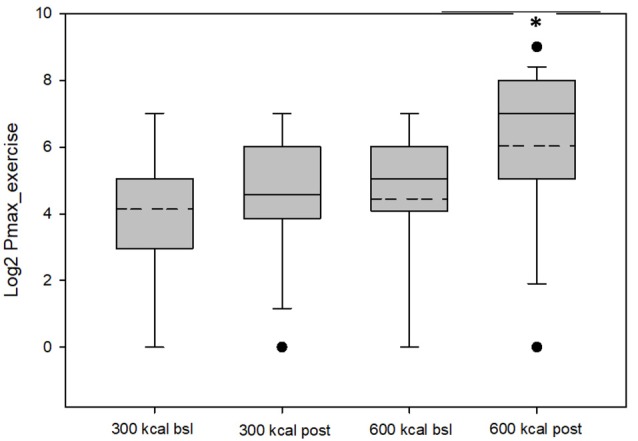
Values for P_max_ exercise (log-transformed) for the 300 kcal per session and 600 kcal per session groups at baseline and post-intervention. The dashed lines represent the mean value, the box represents the interquartile range (25th to 75th percentile), solid line represents the median, and black circles represent outliners, which were included in the analysis and did not change overall results when removed. *Mean P_max_ greater than baseline (*P* = 0.05).

Baseline group data are presented in Table 1. As shown in Table 2 and Figure 1, RRV_exercise_ for both the 600 kcal/session and 300 kcal/session groups increased (*P* < 0.05) after the 12-week intervention. Both groups saw similar decreases (*P* < 0.01) for Pmax_sed_, while only the 600 kcal group increased (*P* < 0.01) their Pmax_exercise_. Both groups saw similar increases (*P* < 0.05) in tolerance and preference + tolerance for exercise intensity after the 12-week intervention while only the 600 kcal group increased (*P* < 0.05) preference for exercise intensity. Changes in RRV_exercise_ were predicted (*R*^2^ = 0.70, *P* < 0.01) by changes in preference and tolerance for exercise intensity (*β* = 0.32, semi partial *R*^2^ = 0.06, *P* < 0.05) when covarying for baseline RRV (*β* = −0.98, semi partial *R*^2^ = 0.57, *P* < 0.01) and baseline preference and tolerance for exercise intensity (*β* = 0.51, semi partial *R*^2^ = 0.08, *P* < 0.01). Subjective ratings of liking of exercise were not correlated with RRV_exercise_ at baseline (*r* = 0.22, *P* = 0.22) or post (*r* = 0.27, *P* = 0.15). The change in liking of exercise also was not correlated (*r* = −0.01, *P* = 0.97) with a change in RRV_exercise_. Covarying for baseline liking scores did not change these results. Changes in physiological measures (body composition, resting metabolic rate) have been reported elsewhere (Flack et al., 2018, 2019) and were not significant predictors of RRV_exercise_, Pmax, or changes in these variables in the present analysis.

**Table 1:** Baseline demographic measures of study participants randomized to exercise interventions of expending 300 or 600 kcal per exercise session, 5 days per week, for 12 weeks.

	300 kcal/session (*n* = 18)	600 kcal/session (*n* = 18)
Age (years)	26.6 ± 5.5	29.4 ± 5.4
Weight (kg)	87.3 ± 16.2	85.5 ± 15.3
Height (cm)	168.2 ± 9.2	169.3 ± 10.6
BMI (kg/m^2^)	30.7 ± 4.3	29.6 ± 3.0
% body fat	37.1 ± 7.9	37.5 ± 6.7

*Data are mean ± SD. BMI, body mass index*.

**Table 2:** Outcome variables at baseline and 12-weeks for participants exercising to expend either 1,500 kcal/week or 3,000 kcal/week for 12 weeks.

	300 kcal/session (*n* = 14)			600 kcal/session (*n* = 15)		
	Baseline	12 week	adjusted group change (95% CI)^1^	Baseline	12 week	adjusted group change (95% CI)^1^
${\text{RRV}}_{\text{exercise}}^{2}$	0.67 ± 0.10	0.88 ± 0.1	0.17 (0.02, 0.31)*	0.78 ± 0.1	0.91 ± 0.1	0.17 (0.03, 0.31)*
P_max_ exercise^3^	35.1 ± 11.0	42.0 ± 10.7	5.3 (−51.3, 61.9)^*,∧^	41.1 ± 10.7	137.6 ± 35.4	98.0 (43.3, 152.7)^*,∧^
P_max_ sedentary^4^	13.4 ± 5.0	5.4 ± 2.6	−6.4 (−9.5, −3.2)*	9.1 ± 4.5	1.9 ± 1.2	−8.7 (−11.8, −5.7)*
Preference for exercise intensity^5^	24.6 ± 1.3	26.0 ± 1.5	1.1 (−1.6, 3.8)	26.1 ± 1.2	29.3 ± 1.5	3.5 (0.9, 6.2)*
Tolerance for exercise intensity^5^	24.4 ± 1.8	28.4 ± 1.4	4.2 (2.4, 6.0)*	23.6 ± 1.5	27.5 ± 1.1	3.7 (2.0, 5.5)*
Preference + Tolerance^5^	48.9 ± 2.8	54.4 ± 2.3	5.3 (1.7, 9.0)*	49.7 ± 2.4	56.8 ± 2.1	7.3 (3.8, 10.8)*

Data are mean ± SE. Data include only those who completed the intervention and all assessments (*n* = 29). ^1^Change score data are adjusted for baseline values, tolerance additionally adjusted for sex. ^2^RRV_exercise_: calculated as P_max_ exercise/(P_max_ sedentary + P_max_ exercise). ^3^P_max_ exercise: the last schedule of reinforcement completed for exercise during the behavioral choice task. ^4^P_max_ sedentary: The last schedule of reinforcement completed for sedentary behaviors during the behavioral choice task. ^5^Preference and tolerance for exercise intensity: assessed via Preference and Tolerance for Exercise Intensity Questionnaire. *Change score different from zero (*P* < 0.05). ^∧^Change scores differ between groups (*P* < 0.05).

## Discussion

There is still a great deal to be learned regarding how to prescribe the number of sessions, frequency of sessions, session dose or energy expenditure, session duration, intensity, and pattern (constant load, interval) to optimally increase RRV_exercise_. Our previous work with a low dose exercise intervention (expending 300 or 150 kcal per session, three times per week for 6 weeks) was not successful in producing greater exercise reinforcement (Flack et al., 2019). Taking guidance from drug abuse literature, exposures to cocaine or amphetamine daily for 10 days (Mendrek et al., 1998) or every 2 days for 20 days (Covington and Miczek, 2001) have been effective at increasing drug reinforcement in rats. Given that drug reinforcement requires dopamine, it is likely that a more frequent or larger total volume of drug use is needed to instigate a dopaminergic response. Based on our and other’s findings noting a similar dopaminergic response to exercise (Rhodes and Garland, 2003; Bronikowski et al., 2004; Knab and Lightfoot, 2010; Flack et al., 2019), we are speculating the sensitization from frequent drug exposures follows a similar pattern in exercise reinforcement, in that more frequent exercise or more total exercise volume (weekly volume/energy expended through exercise) may be an important factor in producing incentive-sensitization of exercise in humans. However, little is known about the neural processes controlling incentive-sensitization for exercise, and it is possible that additional neural mechanisms are in play specific to exercise reinforcement, not following patterns of drug abuse reinforcement. The present results, coupled with our previous work, follow the evidence from drug abuse, indicating that larger doses of structured exercise (5 days per week for 12 weeks) are needed to promote increases in RRV_exercise_ among overweight/obese individuals. RRV_exercise_ increased and responding for sedentary activities decreased in both groups engaging in either 300 or 600 kcal per session, while only the 600 kcal experienced an increase in responding for exercise. Therefore, it appears that 300 kcal (33 min of exercise) per session, 5 days per week is a large enough amount of exercise to increase RRV_exercise._ This increase is primarily driven by decreasing sedentary reinforcement (Pmax_sed_), which is likely to be an important step in becoming more physically active. It is possible that when sedentary people are forced to choose exercise over sedentary behaviors, as in our intervention, their sedentary pursuits become less predominate in their lives and thus lose some of their reinforcing value. However, to instill greater Pmax_exercise_, sessions need to be 600 kcal or approximately 56 min per session, 5 days per week.

Exercise increased both preference and tolerance for exercise intensity discomfort in overweight to obese adults, which extends cross-sectional work demonstrating that individuals who meet activity guidelines have greater tolerance to exercise intensity discomfort (Flack et al., 2017a). This is a welcomed result as many overweight/obese individuals find exercise extremely unpleasant and therefore difficult to maintain (Ekkekakis and Lind, 2006). The increases in, tolerance and preference + tolerance for exercise intensity were not dependent on the doses of exercise tested in the present study, although increases in preference for exercise intensity was only observed in the 600 kcal group. The structure of the exercise sessions may have contributed to the improvements in preference/tolerance for exercise discomfort and RRV_exercise_. The intensity of exercise during the sessions were not self-selected as participants followed prescribed HR based exercise plans that resulted in them meeting their assigned energy expenditure groups. This may have resulted in the participants exercising at a greater intensity and experiencing greater discomfort than if the exercise intensity would have been self-selected (Ekkekakis and Lind, 2006).

Increases in preference and tolerance for exercise intensity predicted increases in RRV_exercise_ when covarying for baseline RRV_exercise_ and baseline preference and tolerance for exercise intensity. The current longitudinal results strengthen and extend previous cross-sectional work that demonstrated preference and tolerance for exercise intensity was positively associated with RRV_exercise_ (Flack et al., 2017a).

It seems reasonable that gaining greater preference and tolerance for exercise intensity associated with exercise is necessary before exercise becomes more reinforcing. Tolerance to the unpleasant aspects of exercise may be more closely associated with the affective responses to exercise than with RRV_exercise_. Changes in liking of exercise were not correlated with changes in, tolerance in the present study; however, affect during exercise was not assessed. An individual that experiences exercise-induced aches and discomfort would be expected to experience negative affect during exercise if they had low tolerance for exercise discomfort. On the other hand, if the individual had greater tolerance, they may derive pleasure from such exercise even when these unpleasant sensations are present. Therefore, preference and tolerance for exercise intensity and RRV_exercise_ could act *via* independent neurobiological systems to promote greater usual PA participation. Indeed, greater frontal electroencephalographic asymmetry, specifically greater left frontal activity relative to right activity, predicts positive affect following exercise (Davidson and Irwin, 1999; Davidson, 2000). In contrast, dopaminergic neurons located in the midbrain structures (substantia nigra and ventral tegmental area) control dopamine release and the reward system that mediates the reinforcing value of behaviors such as food and drugs (Arias-Carrión et al., 2014). With RRV_exercise_ and tolerance acting independently on different neurobiological systems, their influence for PA/exercise participation may be distinct. For instance, RRV_exercise_ may shift behavioral choice towards exercise and away from sedentary alternatives, while increasing one’s tolerance to exercise intensity discomfort could result in greater effect during and after exercise. Both would be expected to improve exercise participation, exercise as a habit, and meeting the PA guidelines (Ekkekakis et al., 2005, 2008b; Flack et al., 2017a,b). It must be stated, however, that the current study did not elucidate specific neurobiological pathways and thus it is not certain of the exact mechanism(s) at play for incentive-sensitization of exercise. It is possible that cognitive processes that work separate from central dopamine metabolism are in play (Chatzisarantis et al., 2008). Improved physical fitness may also influence RRV_exercise_ by allowing individuals to exercise at greater intensities with reduced discomfort, or simply repeating bouts of exercise, may help people better psychologically tolerate exercise discomfort and promote greater RRV_exercise_. Future research may wish to examine if greater tolerance for exercise discomfort derives from other factors related to RRV_exercise_, such as self-efficacy or intrinsic motivation.

This study is not without limitations. As a secondary analysis of a larger study, a control group was not included. It is, therefore, possible that individuals may have increased their RRV_exercise_ apart from the exercise intervention. Assessments of habitual PA were also not included, which would have provided information on actual behavior change and whether increasing RRV_exercise_, preference and tolerance for exercise intensity did indeed result in greater usual PA. However, previous research has demonstrated that these factors are associated with engaging in PA to the amount of meeting activity guidelines (Flack et al., 2017a), suggesting that the changes observed in the current study would have positively influenced participants’ PA. Since participants volunteered for this study, they may have been more motivated to start exercising than the average sedentary individual, which was indeed observed by the greater baseline P_max_ for exercise than for sedentary (i.e., RRV_exercise_ greater than 0.5). Despite this elevated RRV_exercise_ at baseline, individuals still increased RRV_exercise_ after 12 weeks of exercise training. It is likely that greater changes would be observed if less motivated individuals were included (Berntson et al., 1994). Additionally, of the 29 participants who completed the current study, 26 were Caucasian (one American Indian, one multi-racial, and one African American), thus limiting the generalizability to other racial/ethnic groups. Although overweight to obese, these participants were otherwise healthy young adults. It is uncertain whether those with obesity-related comorbidities or older adults can increase RRV_exercise_ and preference/tolerance for exercise discomfort as observed in the current sample. It also may be interesting to compare the normal weight to obese individuals in their ability to increase Pmax or RRV_exercise_ as obesity can alter reward system function (Ziauddeen et al., 2015).

In conclusion, the present study demonstrates that repeated exposures to exercise *via* a structured, exercise program that expends at least 300 kcal/session, performed 5 days per week, for 12 weeks increases the RRV_exercise_ by decreasing the reinforcing value of sedentary alternatives. However, increases in RRV_exercise_ were observed only at a greater dose of 600 kcal/session. Increases in preference and tolerance for exercise intensity predicted increases in RRV_exercise_, possibly pointing to a necessary antecedent for incentive-sensitization of RRV_exercise_ to take place. These psychological and behavioral adaptations to strenuous exercise should increase PA behavior. Future studies would benefit from further investigation of exercise program parameters such as the number of sessions, frequency of sessions, session dose or energy expenditure, session duration, intensity, and pattern (constant load, interval) that most effectively improve incentive-sensitization of exercise and the tolerance and preference for exercise intensity.

## Data Availability Statement

The Grand Forks Human Nutrition Research Center Data Access Committee, an entity of the United States Department of Agriculture, Agricultural Research Service, can be contacted by interested researchers inquiring about gaining access to all data presented.

## Ethics Statement

The studies involving human participants were reviewed and approved by University of North Dakota Institutional Review Board. The patients/participants provided their written informed consent to participate in this study.

## Author Contributions

KF and JR designed the study. KF and KU collected the data. KF, KU and LJ analyzed the data. KF drafted the manuscript with substantial contributions from KU, LJ and JR. All authors have approved the final version.

## Conflict of Interest

The authors declare that the research was conducted in the absence of any commercial or financial relationships that could be construed as a potential conflict of interest.
